# Research protocol of a multifaceted, comparative mixed-method study: Young people transitioning from out-of-home care in Norway and Australia - Interrelationships between policies, pathways, and outcomes

**DOI:** 10.1371/journal.pone.0323948

**Published:** 2025-05-15

**Authors:** Veronika Paulsen, Christian Wendelborg, Stian H. Thoresen, Lauren Parsons, Donna Chung, Melissa O’Donell, Regine Ringdal, Reinie Cordier

**Affiliations:** 1 Diversity and Inclusion, NTNU Samfunnsforskning AS, Trondheim, Norway; 2 Curtin School of Allied Health, Faculty of Health Sciences, Curtin University, Perth, Australia; 3 Australian Centre for Child Protection, University of South Australia, Perth, Australia; 4 Department of Social Work, Education and Community Wellbeing, Faculty of Health and Life Sciences, Northumbria University, Newcastle, United Kingdom; PLOS: Public Library of Science, UNITED KINGDOM OF GREAT BRITAIN AND NORTHERN IRELAND

## Abstract

**Introduction:**

Young people who have been in the care of child welfare systems face myriad challenges as they transition into adulthood, resulting in poor long-term outcomes. The overwhelming international trend shows that compared with their peers who have not been in care, care leavers are more likely to experience unstable living conditions, low engagement in employment, financial instability, low levels of educational attainment, poor physical and mental health, and involvement in criminal justice systems. Child welfare policies to support young people as they transition to independence are implemented differently in different countries, leading to different outcomes.

**Methods and analysis:**

We will compare administrative data from Norway and Australia to objectively quantify and compare, at a population level, associations between service use pathways and young people’s outcomes as they transition from out-of-home care within different policy contexts. In addition, the study includes in-depth interviews with young care-experienced people, their carers, and service providers. The mixed-methods study will make cross-country comparisons of the lived experiences of young people, their carers, and service provision practices that act as barriers and facilitators to positive long-term outcomes within Norway and Australia. The combined findings from the population-level data and an in-depth understanding of their lived experiences will identify a best practice model to facilitate better outcomes. A critical policy analysis and synthesis will be conducted alongside these studies to highlight the influence of policy on service provision practices, young people’s pathways, and outcomes.

**Discussion:**

To date, there have been no international comparisons at a population level to determine how various policy contexts influence outcomes for care leavers, thus not allowing a granular explanation for why young people continue to experience poor outcomes. This project will develop a nuanced and dynamic understanding of how different policies and practices produce pathways that either promote or constrain positive outcomes in adulthood. The comparisons across Norway and Australia will generate unique knowledge by enhancing our understanding of internal and external child welfare services, country-specific, and individual characteristics in policies and practices enhancing outcomes for young people transitioning from out-of-home care.

## Introduction

Children and young people may enter out-of-home care (OHC) for various reasons, and care-experienced young adults are a diverse and heterogeneous group. Many care-experienced young adults develop rich and meaningful lives with a wide range of social, political, and professional roles. This includes a growing cohort of care-experienced researchers who are increasingly setting the research agenda, as well as care-experienced people who are contributing towards, or even spearheading, social and political initiatives to better outcomes for care leavers. Nevertheless, there is overwhelming international evidence that young people who have been in the care of child welfare services (CWS) experience poorer long-term outcomes [1–7] at rates disproportionate to their peers in the general population.

The poorer long-term outcomes appear to be a consistent trend internationally, regardless of differing policy contexts across countries [3,8]. International policy variations are well recognised; however, how these differing policy contexts precisely influence long-term outcomes and whether any support systems funded under various policies facilitate more positive transitions to adulthood are under-researched. Rather, international comparisons have largely focused on rates and characteristics of children and young people entering OHC [e.g., 9,10]. As such, research is needed to generate nuanced and dynamic knowledge of how different child welfare policy contexts impact long-term outcomes for care leavers. Establishing an evidence base will require international comparisons accounting for both population-level outcomes and individual experiences and contexts.

The Norwegian Research Council funds this project for the period 2021–2025 and will compare CWS in Norway and Australia. While the social policy approaches and provision of child protection services vary between these countries, consistent with international research generally, poor long-term outcomes into adulthood have been reported in both countries [2,5]. The Norwegian CWS is described as a ‘family service system’ with a relatively low threshold for providing a range of family services [11]. The system focuses on early intervention, prevention, and support and has statutory responsibility for child protection monitoring and intervention. The Australian child protection systems are predominantly tertiary responses to child harm. As a Federal system, each State and Territory is responsible for its own child welfare service. While the importance of early intervention is recognised, in practice, most services are initiated following reports of maltreatment with Aboriginal families over-represented in child protection [12].

### Prevalence of young people in OHC

In 2022, 47,034 Norwegian children and young people were involved in the CWS, with 82% receiving supportive measures. The remaining 18% (8,249) were placed in OHC, equating to a rate of 7.4 children in OHC per 1,000 [13]. In comparison, 45,300 Australian children were in OHC on the 30^th^ of June 2023, a rate of 7.9 per 1,000 [14]. When considering only children in OHC, Norway and Australia have similar placement rates, but the Norwegian CWS is much more comprehensive.

### Placement types

The most common placement types are family-based community placements. Among the 11,141 children and young persons in OHC in Norway on the 31^st^ of December, 2022, 28% were in kinship care, 63% were in foster care, while the remaining 9% were in residential care [13]. In Australia, 89% of children in OHC are in home-based care, which includes mostly relative or kinship care (54%) and foster care [39%, 14]. Approximately 9.5% of young people in OHC live in residential care, which is mainly used in situations where children have complex needs [14]. Thus, while the OHC placements rate is similar, the proportion of placement types and how these are utilised may be somewhat different in Norway and Australia.

### Period of after-care

Norwegian legislation states that young people receiving CWS support before 18 years old can continue to receive support until they are 25 years old. Of young people who have been placed in OHC, around 70–80% access after-care services; however, most do not receive support beyond the age of 20 or 21 years [5]. In Australia, the statutory responsibility of child protection authorities for young people in care ends upon a person reaching 18 years of age. Until recently, young people had access to after-care support after 18 years via non-government organisations until the age of 25 years. These leaving care services positively impacted young people’s long-term outcomes; however, only a third (34%) of eligible young people were utilising the services [15]. A range of policy, budgetary, and legislative reforms enacted across all Australian jurisdictions between 2018 and 2023 has seen the extension of care arrangements to the age of 21 years for eligible young people in some states and for all young people in other states [16]. The Australian branch of this project is situated in Western Australia (WA), where universal extended care (Home Stretch WA) commenced implementation in 2021, and young people are provided with the option to continue to receive support until the age of 21 years [17].

### Policy implementation

In Norway, child welfare policy and legislation are the national government’s responsibility, with local authorities (municipalities) responsible for investigation and service provision. The legislation and guidelines are very broad, enabling considerable discretion in implementation, leading to large variations in practices between municipalities and regions. While a national framework sets out high-level outcomes and strategies, child protection policy, legislation and services within Australia are the responsibility of each State or Territory Government in the federal system of government, with variability in practice across Australia.

The research protocol described in this paper will give a detailed description of the research plan for the project “*Young people transitioning from out-of-home care in Norway and Australia: Interrelationships between policies, pathways, and outcomes*”. The project will focus on the pathways leading to children being placed in state care, seminal change points and pathways while in care, engagement of the family of origin, and exit pathways and post-care outcomes. The overall project objective is to expand the knowledge of the impact of social welfare policy on long-term outcomes for young care-leavers and identify opportunities for service provision and practice improvements to redress poor long-term outcomes for young people post-care. The project consists of four studies (studies A-D) guided by four interrelated research questions:

1. How do critical CWS policies in Norway and Australia influence pathways and outcomes post-care (Study A)?
2. What are the population-based post-care pathways and outcomes for care leavers in Norway and Australia (Study B)?
3. What are the transition from care and post-care lived experiences of care leavers, their carers, and their service providers (Studies C & D)?
4. What policy and practice recommendations and service provision responses will improve outcomes, remove barriers, and facilitate positive transition pathways from care to independence for care-leavers in Norway and Australia (Studies A-D)?

## Materials and methods

To shed light on the research questions, a mixed-methods design, comprised of four distinct but related studies, will be used to optimise understanding of the relationships between policies and outcomes for young people transitioning from OHC. The focus is on young people aged between 15 and 27 years who are in care or have recently left care and may be transitioning to independent living or (biological) family reunification. An international advisory board is guiding the research across the project’s lifespan. In addition, a local advisory board has been established in each jurisdiction. The Australian arm of this study is an extension of a Western Australian study of transitioning from OHC: Navigating Through Life [cf. 18].

### Study design

**Fig 1:**
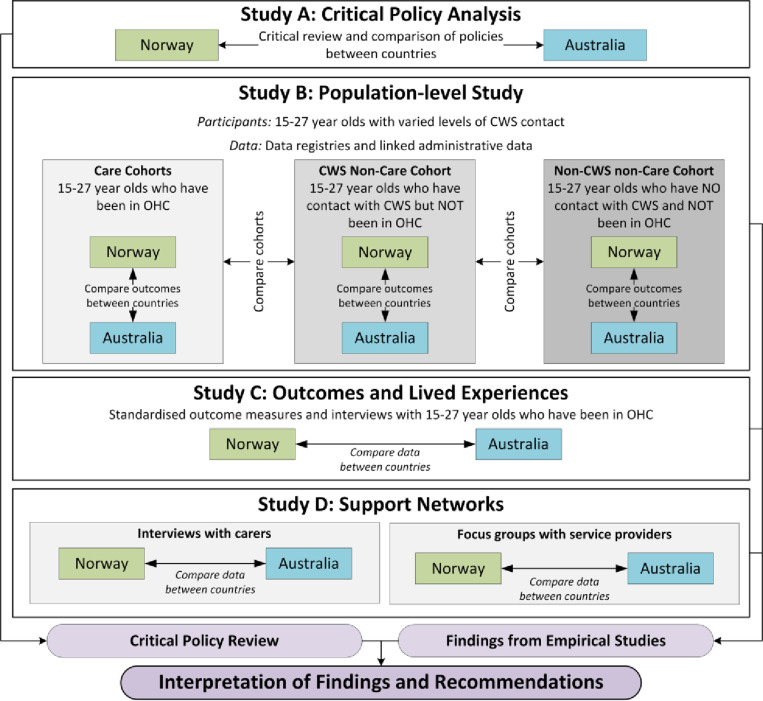
Study design.

The project started in June 2021 and ends in December 2025. Each of the four studies will utilise data from a range of sources, and the studies are integrated both horizontally (between countries) and vertically (between studies). Findings from Study A (a critical policy analysis), combined with those from the empirical studies (Studies B-D), will be used to contextualise the project’s findings within a policy framework and make recommendations for future policy and practice improvements. The relationships between the studies are illustrated in Fig 1, and each study’s details are described.

### Study A: Critical policy analysis

*Study A* is a critical policy analysis of Norwegian and Australian OHC policies (RQs 1 & 4) drawing on Bacchi’s approach: *What’s the problem represented to be?* (WPR) [19]. The WPR approach involves an ongoing and iterative process of critical review of policies guided by the following six questions:

1. What’s the ‘problem’ represented to be in the respective child welfare policies?
2. What presuppositions or assumptions underlie this representation of the ‘problem’?
3. How has this representation of the ‘problem’ come about?
4. What is left unproblematic in this problem representation? Where are the silences? Can the ‘problem’ be thought about differently?
5. What effects are produced by this representation of the ‘problem’?
6. How/where has this representation of the ‘problem’ been produced, disseminated and defended? How could it be questioned, disrupted, and replaced?

The results will inform the critical analysis of child welfare policies, leading to evidence-based policy and practice recommendations as a major project outcome. The critical policy analysis will provide a richer understanding of underlying assumptions; interconnected challenges; and social, welfare, and cultural contexts of child welfare policy within Norway and Australia, which is central to understanding how different policies and their contexts affect young people’s pathways and outcomes as they transition from OHC.

A critical analysis using Bacchi’s WPR approach will be conducted using selected white papers and supporting documentation published by the Norwegian and Australian governments dealing with specific child protection and transition from OHC issues. We will also critically analyse publications relevant to the policies in the two countries and scholarly research articles that point out factors that may influence pathways and outcomes for care leavers, in addition to pilot projects that have been initiated to support the development of, or as a response to, these policies.

The critical analysis will identify similarities and differences in the policies, underlying assumptions; interconnected challenges; and social, welfare, and cultural contexts of child welfare policy, which, based on previous research, can impact pathways and outcomes in the different countries. The findings will be used to identify focus areas and the analytical framework for subsequent studies, ensure that policy implications are included in all studies, and develop suggestions for improving policy and practice implications. This will create a set of key outcomes to identify at a population level (Study B); key transitions, pathways, and turning points to document among the lived experiences of care-leavers (Study C); and processes, barriers, and facilitators in service responses (Study D).

### Study B: Population-level study of pathways and outcomes through administrative data

We will use administrative data from Norway and Australia to examine population-level outcomes and pathways for young people who have or are transitioning from OHC. Utilising linked administrative data will enable the examination of between-country differences in CWS and reveal distinct policy-related outcomes and pathways for comparison.

#### Participants.

Study B involves three cohorts: The **OHC Cohort** will comprise all young people born in Norway or Western Australia (Australian jurisdiction with the most comprehensive data available) between 1994 and 2006 (i.e., aged 15–27 years at study commencement), who have been placed in OHC. Two comparison groups will be sourced from administrative data within each country. Comparison groups comprise firstly young people who have had CWS contact but have not been placed in OHC (CWS Non-Care Cohort), and secondly, young people who have not had CWS contact and have not been in OHC (Non-CWS Non-Care Cohort). These will enable within- and between-country comparisons of outcomes for care leavers to those who have had contact with CWS but were not in care, as well as those from the general (non-CWS non-Care Cohort) population to identify factors that foster successful outcomes.

#### Data sources.

Outcome data will be derived for the respective cohorts from administrative records across five domains: 1) *education* (e.g., attendance, attainment, suspensions); 2) *mental and physical health* (e.g., diagnoses and service interactions, length of stay, self-harm episodes, suicide, hospital admissions, emergency department presentations); 3) *employment status* (e.g., rates of unemployment/under-employment, welfare dependency); 4) *access to welfare and social security benefits* (e.g., supplementary benefits, after-care services, public and social housing); and 5) *justice system involvement* (e.g., persons charged, criminal sanctions, imprisonment, sequence of offending, and pathways through justice system). The Norwegian and Australian data linkage systems allow for genealogical linkage, enabling the investigation of parental factors that may impact children’s pathways.

Several national population‐based registries in **Norway** will be utilised. These registers have encrypted person-identifiable records that allow us to identify and link persons across registers. Each person-identifiable record also has a family-identifiable record, allowing easy attainment of background and family variables. The child welfare statistics register (Statistics Norway [SSB]) will be used to identify the OHC and CWS Non-Care Cohorts and contain detailed longitudinal data about measures received from CWS from 1993 onwards. These records will be linked to other registers and sources (e.g., FD-Trygd, National Education Database, Norwegian Patient Registry) to identify pathways and outcomes.

Administrative records within **Australia** are decentralised, with population-based data collections across states and government departments and services within each state. Data linkage methods are used to bring together data on the same person from different sources. WA data will be sourced from the linked, administrative data system created and maintained by WA Government. An application through the WA Government to access de-identified data has been made. Data are linked using deterministic and probabilistic methods and a best practice separation model (Table 1).

**Table 1:** Datasets and outcomes derived from administrative data.

Outcome areas	Databases/registers in Norway	Databases/registers Australia	Operationalised within Norway	Operationalised within Australia
Justice	Crime and Justice System (Statistic Norway)	Police Offences, Police Move on Notices, Youth Justice, Adult Corrections, Custodial Stays	Offense Misdemeanour/Felony Number of Offenses	Age of offences, sequence of offending and pathways through the justice system. Types of offences and orders, diversionary processes, incarceration.
Education	The National Education Database (Statistic Norway)	School Enrolments, Literacy and Numeracy Assessment Data, Student Attendance, Student Suspensions	School achievement, area of study, completion of upper secondary school, higher education data	Literacy and numeracy achievement, school attendance and suspensions.
Health/substance use/Mental health	Norwegian Patient Registry (Norwegian Institute of Public Health) Cause of Death Registry (Norwegian Institute of Public Health) Municipal Patient and User Registry (Norwegian Institute of Public Health) Population register (Statistic Norway) The Armed Forces Health Registry (The Armed Forces)	Hospital Morbidity Database, Emergency Department Data, Death Register, Mental Information System, Birth Registrations Midwives Notifications	Hospital and emergency visits: Timing, frequency, length of stay, major diagnoses, injury causes. Substance use: Interdisciplinary Specialized Addiction Treatment, Drug/alcohol-related diagnoses. Mental health care contacts: Timing, frequency, diagnoses, co-morbid issues, self-harm, suicide. Pregnancy contacts, age at birth, pregnancy complications, birth outcomes, mental health contacts, substance use contacts.	Hospital and emergency visits: Timing, frequency, length of stay, major diagnoses, injury causes. Substance use: Drug/alcohol-related diagnoses, police/justice contacts for substance issues or violence. Mental health contacts: Timing, frequency, diagnoses, co-morbid issues, self-harm, suicide. Pregnancy contacts, age at birth, pregnancy complications, birth outcomes, mental health contacts, substance use contacts.
Child Welfare Services and Welfare	FD-Trygd (Statistics Norway’s events database) Child welfare statistics (Statistic Norway) Housing Conditions Registry (Statistics Norway)	Child protection clients, Child protection notification, Child protection investigations and substantiations, Child protection orders, Child protection periods of care, Public housing	Social conditions, Social Security pensions, after-care, Housing conditions	Child protection contacts, time in OHC, type of OHC, applications for public housing, public housing tenancies.
Employment/ income	Labour market and earnings (Statistic Norway)	NA	Employment statistics, labour-market measures, income	NA

In Norway, the linking key is the personal identification number, allowing all information about gender, year of birth, residence, relocations, and immigration status to be centrally registered, eliminating the need to retrieve it from various databases/registers. NA = Not applicable to Western Australian linked data as the Australian Federal Government holds this data.

#### Analysis.

Multiple regression analysis to determine factors that predict both positive and negative outcomes for young people in OHC within and between countries. Potential predictors may include the reason for placement in OHC, age at the time of placement, placement stability, type of placement and service access. Where appropriate, conditional logistic regression will be used to account for matching. More advanced statistical modelling, such as Latent Class Growth Analysis (LCGA), will be employed to identify the characteristics of subgroups of young people who followed distinct trajectories within and between countries. Group-based trajectory modelling will be conducted to identify factors associated with outcomes of interest. Path analysis will be used to explore the mediating and moderating factors associated with outcomes of interest.

### Study C: Cross-sectional mixed methods study with young people in and transitioning from OHC

*Study C* is a mixed-method study of a subset of young people transitioning from care (15–27 years) that extends and complements Study B (RQ 3). A nuanced understanding of young people’s lived experience of transitioning from care will be pursued, complemented with psychosocial outcomes, pathways, and trajectories. Young people will identify challenges associated with transitioning from care and the service responses that have acted as barriers and facilitators of positive transitions.

#### Participants.

A sample of 80 care-experienced young people from Norway and Australia will be recruited based on the following inclusion criteria**:** aged 15–27 years; in or recently left OHC; and able to consent to participate in the study. We will strive for maximum variation in the sample regarding gender, ethnicity, age, in care or left care, type of placement (foster home, institution) and young people’s background and potential challenges (cognitive difficulties, minority background, low income/ socio-economic status, substance abuse problems and mental difficulties). Researchers within each country will use existing collaborations with CWS and other non-government organisations to recruit participants.

#### Data collection.

Both quantitative and qualitative data will be collected from young people, involving standardised outcome measures and interviews. The recruitment started in January 2023 and the tentatively end date of recruitment is August 2025. Standardised outcome measures will be administered to identify key dimensions of young people’s lives to enable a more granular exploration of young people’s wellbeing that cannot be captured through administrative service data in Study B (see Table 2).

**Table 2:** Standardised outcome measures.

Outcomes	Measures	Languages
1. Mental health and wellbeing	Strengths and Difficulties Questionnaire in Norway [20] and Strong Souls in WA [21]	Norwegian and English
2. Social inclusion*	Measures developed for the current WA study	Norwegian and English
3. Independent Living Skills (ILS)*	Measures developed for the current WA study	English
4. Self-Determination	AIR Self Determination Scale [22]	English
5. Adverse Childhood Events	ACEs Questionnaire [23]	English

*The Social Inclusion and ILS measures are being validated and capture participation and sense of belonging in different settings as well as skills needed for independent living.

Interviews with young people will focus on the lived experiences of transitioning into adulthood to capture the following topic areas: living arrangements, education, employment, social and familial connections, social supports, health and wellbeing, attachment, opportunities and barriers to engaging with services, and exploration of helpful and unhelpful service delivery encounters. The qualitative interviews will be audio-recorded and transcribed.

The interview schedule and outcome measures have been translated into Norwegian and have been piloted with a small number of care-experienced young people and young people in OHC in Norway (N = 8). The Norwegian translation of these outcome measures (ILS, AIR Self Determination Scale, and Adverse Childhood Events) will be validated as part of the larger study.

#### Data analysis.

The sample size is sufficient for varied regression analysis of significant variables influencing outcomes. Hierarchical Bayesian regression models will analyse outcome measure data as they are not sample size dependent. These models will allow robust estimation of treatment effects, even in smaller (national) subgroups, by effectively borrowing information from other sub-groups when estimating sub-group specific responses [24]. In addition, we will use a second hierarchical level to simultaneously estimate effects on multiple outcomes (e.g., self-determination and mental health and wellbeing), improving effect estimates by borrowing information between outcomes. To optimise the available information, all analyses of rating scale data will use Item Response Theory modelling [Rasch analysis; 25].

Interview transcripts will be analysed thematically according to common qualitative data analysis practises, which include data reduction, meaning-making through data coding, and analysis and verification of the identified themes [26–28]. Textual analysis will be utilised using computer software. The qualitative data analyses will draw on life course theory (LCT) [29] to distinguish ways in which a person’s experiences may have been influenced at various points over time. These findings will contextualised alongside the quantitative outcome measures by being mapped against pathways, transitions, key turning points and spheres of influence drawing on LCT [29].

### Study D: Carers and service provision responses for positive transitions from care

*Study D* is a qualitative study capturing the perspectives of practitioners and carers (RQ 3) who provide services to young people transitioning from care and carers (e.g., foster carers and residential care staff). The study will identify current and future service responses to address barriers and promote positive transitions identified by young people in Study C (RQ 4).

#### Participants.

Practitioners working with young people transitioning from care will be invited to participate in focus group interviews (five in each country). We will use maximum variation within the sample to obtain perspectives across a range of service categories, including OHC transition and after-care services, young people services (e.g., homelessness, legal, health, mental health, alcohol and drug, employment, justice), and adult services (e.g., homelessness, mental health, financial assistance, community housing, police, and disability).

Carers of young people will also be invited to participate in focus groups or individual interviews to explore their perspectives on the challenges, barriers, and facilitators for positive transitions from OHC. They may be the carers of Study C participants or others experienced in caring for young people in OHC. Forty carers will be recruited from each country, and the study aims to provide maximum variation within the sample to obtain perspectives across different caring arrangements (e.g., foster, kindship and paid residential carers). The research team will leverage existing relationships with service providers and carers associations or networks to recruit participants.

#### Data collection.

Focus groups and individual interviews will examine pathways into and out of the agencies and/or care arrangements, emphasising service availability and gaps, as well as young people with complex and multiple needs. The recruitment started in May 2024 and the tentatively end date of recruitment is August 2025. Barriers and facilitators to positive transitions from care will be emphasised to examine how current service responses to young people vary individually and structurally across jurisdictions. In addition, alternative service system responses and future solutions will be elicited from the groups to highlight the service system factors influencing different outcomes.

#### Data analysis.

Transcripts will be analysed as described for Study C interview data.

### Ethical considerations

All studies within the project have obtained appropriate approvals from all relevant Human Research Ethics guidelines: The Norwegian Agency for Shared Services in Education and Research, the Regional Committee for Medical and Health Research (REC) in Norway (application number 615022), and Curtin University’s Human Research Ethics Committee (HREC) in Australia (HRE2018–0170). Following institutional HREC approval, applications to access Western Australian linked data was made to the data custodians of each dataset, and additional HREC approval was sought from the WA Department of Health (RGS0000003752). Critical to the Australian context, approval was sought from the Western Australian Aboriginal Health Ethics Committee (approval #1046) to use Aboriginal identifiers in data analyses.

All person-level data will be securely managed and stored in line with best practice separation principles (i.e., all name-identified data will be kept separate from de-identified service level data). Mappings between personal information and service-level data will be created and managed by authorised personnel only. Personal data will be stored within the jurisdiction in which it was collected.

Only de-identified administrative data will be supplied to the research teams in Study B. Data across all studies will be stored and transferred securely and aligned with each county’s data protection and privacy laws. All Studies C and D participants will provide verbal or written consent to participate. Consent for Australian participants <18 years of age (i.e., young people in care) has been provided by the CEO of the Department of Communities (i.e., their legal guardian). At the same time, Australian participants <18 years of age will provide their own consent as mature minors if deemed competent (Gillick competence principle [30]). Norwegian participants between 16–18 years of age will provide consent as mature minors (Norwegian Agency for Shared Services in Education and Research assessment), while parents/guardians of Norwegian participants under 16 years of age will have to provide consent with the youth providing assent. The informed consent will be obtained written or verbal (with audio recording).

## Discussion

This research will increase understanding of how policies and programs influence outcomes from which more effective evidence-based service pathways associated with positive outcomes can be implemented. Despite disparate CWS approaches, there are multiple reasons why the evidence generated from comparing these two countries will identify policies and practices that lead to successful outcomes and transitions from OHC. Firstly, both Norway and Australia have robust government registries of administrative data and data-linkage capacities. This will enable identifying outcomes and relationships at a population level, which is rare internationally. Secondly, both countries recognise poor outcomes among care leavers and have various policies and programs to redress inequities. This will facilitate a rich policy and practice analysis to identify facilitators. Thirdly, while both countries are advanced economies, interesting sociodemographic differences provide a broad spectrum of variables for comparisons (e.g., differences and similarities in migration trends, Indigenous populations, geographical remoteness, and labour market dynamics). This comparison will generate unique knowledge that two single-country studies cannot generate, as the comparisons across jurisdictions provide a greater insight into internal and external CWS, country-specific, and individual characteristics in policies and practices enhancing outcomes for young people transitioning from OHC.

The contextualisation of CWS policy analysis will allow for more detailed explanations, identification of turning points in the lives of young people and their families, and mapping the impact of critical child protection decisions differentiated for different groups of young people in care. The comparison between Norway and Australia and their differential policy contexts will shed light on policy and practice impacts on outcomes at various stages in a child’s journey through CWS’ care.

The translatable findings will provide new evidence about effective policy and practice. This project will develop new knowledge on the relationship between child welfare policies and practices on long-term outcomes for young people who have been placed in OHC. International comparisons in CWS research are rare, and this will be the first robust international comparison of administrative population-based outcomes for care leavers complemented by interview, focus group, and survey data from care leavers and carers. A policy analysis and cross-country comparisons between Norway and Australia will further contextualise these findings. Thus, the potential for substantial impact lies in the integration of these components, which will:

1. Combine rich population-based data from Norwegian and Australian administrative data registries to elucidate, in detail, service use and long-term outcomes for young people exiting OHC.
2. Compare detailed pathways and outcomes for care-experienced young people to highlight the impact of policy on pathways and outcomes.
3. Determine practices within services that act as barriers and facilitators to positive long-term outcomes for care leavers from the perspectives of young people, carers, and practitioners.
4. Document social, cultural, economic, and individual factors that increase the likelihood of better outcomes for young people post-care.
5. Compare and contrast outcomes, barriers, facilitators, and pathways across policy contexts. As such, this research will pave the way towards understanding how variations in policy influence young people’s transitions from care and longer-term outcomes.

This project will facilitate improved CWS policy and practice and ultimately improve life outcomes for young people post-care. The project will make an important contribution given the longstanding and consistent finding that young people in care have poorer outcomes in adulthood compared with the general population. Identification of the key challenges care-leavers experience, including pathways and turning points, will have international significance when aligning these with evidence-based policy and practice that can improve outcomes and ensure that care-leavers are not ‘left behind’ in their transition to adulthood. Seeing the work of such influences over time has considerable implications for practice and policy, given the potential to begin to discern and highlight practical ways in which educational experiences have been shaped.

## Abbreviations

CWS: child welfare services
HREC: Human Research Ethics Committee
ILS: independent living skills
LCT: life course theory
OHC: out-of-home care
REC: Regional Committee for Medical and Health Research
RQ: research question

## References

[1] Mann-Feder VR, Goyette M. Leaving care and the transition to adulthood: International contributions to theory, research, and practice. Oxford University Press; 2019.

[2] Mendes P, Snow P. Young people transitioning from out-of-home care: International research, policy and practice. Springer; 2016.

[3] van Breda ADP, Frimpong-Manso K. Leaving care in Africa. Emerging Adulthood. 2020;8(1):3–5. doi: 10.1177/2167696819895398

[4] Kääriälä A, Hiilamo H. Children in out-of-home care as young adults: a systematic review of outcomes in the Nordic countries. Children and Youth Services Review. 2017;79:107–14. doi: 10.1016/j.childyouth.2017.05.030

[5] Paulsen V, Wendelborg C, Riise, Berg B, Tøssebro C. “Aftercare - a good transition to adulthood?” (Ettervern - en god overgang til voksenlivet?). Trondheim: NTNU Social Research; 2020.

[6] Paulsen V, Thoresen SH, Wendelborg C. Outcomes in adulthood among former child welfare services recipients: findings from a Norwegian registry study covering two decades. Eur J Soc Work. 2022;26(3):411–27. doi: 10.1080/13691457.2021.2016646

[7] Martin R, Cordier R, Jau J, Randall S, Thoresen S, Ferrante A, et al. Accommodating transitions: improving housing outcomes for young people leaving OHC. AHURI Final Report 2021;364.

[8] Stein M. Young people’s transitions from care to adulthood: international research and practice. Jessica Kingsley Publishers; 2008.

[9] Healy K, Lundström T, Sallnäs M. A Comparison of Out-of-home Care for Children and Young People in Australia and Sweden: Worlds Apart? Australian Social Work. 2011;64(4):416–31. doi: 10.1080/0312407x.2011.603092

[10] Tilbury C, Thoburn J. Children in out-of-home care in Australia: International comparisons. Children Australia. 2008;33(3):5–12. doi: 10.1017/s1035077200000262

[11] Berrick JD, Peckover S, Pösö T, Skivenes M. The formalized framework for decision-making in child protection care orders: A cross-country analysis. Journal of European Social Policy. 2015;25(4):366–78. doi: 10.1177/0958928715594540

[12] Child protection Australia: 2018–19. Child welfare series no. 72. Cat. no. CWS 74.

[13] Child Welfare. Children receiving measures from the Child Welfare Services. Available from: https://www.ssb.no/en/sosiale-forhold-og-kriminalitet/barne-og-familievern/statistikk/barnevern

[14] Child Protection in Australia 2022-23. Available from: https://www.aihw.gov.au/reports/child-protection/child-protection-australia-insights/contents/about

[15] Office of the Auditor General Western Australia: Young People Leaving Care; 2018.

[16] Mendes P. “The most significant child welfare reform in a generation”: An examination of the strategies used by the Home Stretch campaign. Aust J Social Issues. 2023;59(2):328–43. doi: 10.1002/ajs4.288

[17] Home Stretch WA; 2024. Available from: https://www.wa.gov.au/organisation/department-of-communities/home-stretch-wa

[18] Parsons L, Chung D, Cordier R, Hodgson D, Lund S, Mendes P, et al. Research protocol of a multifaceted prospective mixed-method longitudinal study: Navigating Through Life - Western Australian study of transitions from out-of-home care. BMC Public Health. 2020;20(1):1180. doi: 10.1186/s12889-020-09138-x 32727442 PMC7389677

[19] Bacchi CL. Analysing policy: what’s the problem represented to be? 1st ed. Frenchs Forest NSW: Pearson Education; 2009.

[20] Goodman R. Psychometric properties of the strengths and difficulties questionnaire. J Am Acad Child Adolesc Psychiatry. 2001;40(11):1337–45. doi: 10.1097/00004583-200111000-00015 11699809

[21] Menzies School of Health Research. Strong Souls assessment tool. Available from: https://www.menzies.edu.au/page/Resources/Strong_souls_assessment_tool/

[22] Wolman J, Campeau P, Dubois P, Mithaug D, Stolarski V. Air self-determination scale and user guide. Palo Alto, CA: American Institute for Research; 1994.

[23] Felitti VJ, Anda RF, Nordenberg D, Williamson DF, Spitz AM, Edwards V, et al. REPRINT OF: Relationship of Childhood Abuse and Household Dysfunction to Many of the Leading Causes of Death in Adults: The Adverse Childhood Experiences (ACE) Study. Am J Prev Med. 2019;56(6):774–86. doi: 10.1016/j.amepre.2019.04.001 31104722

[24] Greenland S. An introduction to instrumental variables for epidemiologists. Int J Epidemiol. 2000;29(4):722–9. doi: 10.1093/ije/29.4.722 10922351

[25] Magno C. Demonstrating the difference between classical test theory and item response theory using derived test data. Int J Educ Psychol Assess. 2009;1(1):1–11.

[26] Miles M, Huberman A. Qualitative data analysis. 2nd ed. Thousand Oaks, London, and New Delhi: Sage Publications; 1994.

[27] Braun V, Clarke V. Using thematic analysis in psychology. Qualitative Research in Psychology. 2006;3(2):77–101. doi: 10.1191/1478088706qp063oa

[28] Boyatzis RE: Transforming qualitative information: Thematic analysis and code development. Thousand Oaks: Sage; 1998.

[29] Elder GH Jr. The life course as developmental theory. Child Dev. 1998;69(1):1–12. doi: 10.1111/j.1467-8624.1998.tb06128.x 9499552

[30] Bart A, Hall GA, Gillam L. Gillick competence: an inadequate guide to the ethics of involving adolescents in decision-making. J Med Ethics. 2024;50(3):157–62. doi: 10.1136/jme-2023-108930 37169548

